# All-Cellulose Composite Laminates Made from Wood-Based Textiles: Effects of Process Conditions and the Addition of TEMPO-Oxidized Nanocellulose

**DOI:** 10.3390/polym14193959

**Published:** 2022-09-22

**Authors:** Eija-Katriina Uusi-Tarkka, Jaka Levanič, Henrik Heräjärvi, Nawar Kadi, Mikael Skrifvars, Antti Haapala

**Affiliations:** 1 School of Forest Sciences, Faculty of Science and Forestry, University of Eastern Finland, FI-80101 Joensuu, Finland; 2 Biotechnical Faculty, Department of Wood Science and Technology, Jamnikarjeva 101, 1000 Ljubljana, Slovenia; 3 Natural Resources Institute Finland, FI-80130 Joensuu, Finland; 4 Department of Textile Technology, Faculty of Textiles, Engineering and Business, University of Borås, S-50190 Borås, Sweden; 5 Swedish Centre for Resource Recovery, Faculty of Textiles, Engineering and Business, University of Borås, S-50190 Borås, Sweden; 6 FSCN Research Centre, Mid Sweden University, SE-85170 Sundsvall, Sweden

**Keywords:** biocomposite, NaOH-urea, solvent system, TEMPO-oxidized nanocellulose, wood fiber

## Abstract

All-cellulose composites (ACCs) are manufactured using only cellulose as a raw material. Biobased materials are more sustainable alternatives to the petroleum-based composites that are used in many technical and life-science applications. In this study, an aquatic NaOH-urea solvent system was used to produce sustainable ACCs from wood-based woven textiles with and without the addition of TEMPO-oxidized nanocellulose (at 1 wt.-%). This study investigated the effects of dissolution time, temperature during hot press, and the addition of TEMPO-oxidized nanocellulose on the mechanical and thermal properties of the composites. The results showed a significant change in the tensile properties of the layered textile composite at dissolution times of 30 s and 1 min, while ACC elongation was the highest after 2 and 5 min. Changes in hot press temperature from 70 °C to 150 °C had a significant effect: with an increase in hot press temperature, the tensile strength increased and the elongation at break decreased. Incorporating TEMPO-oxidized nanocellulose into the interface of textile layers before partial dissolution improved tensile strength and, even more markedly, the elongation at break. According to thermal analyses, textile-based ACCs have a higher storage modulus (0.6 GPa) and thermal stabilization than ACCs with nanocellulose additives. This study highlights the important roles of process conditions and raw material characteristics on the structure and properties of ACCs.

## 1. Introduction

Environmental concerns drive developmental efforts to create sustainable bioproducts, replacing petrochemical plastics with renewable and sustainable resources [1]. All-cellulose composites (ACCs) are a family of single-polymer composites produced from various cellulosic raw materials, such as wood pulp, plant fibers, cellulose nanofibers, microcrystalline cellulose, and wood-based textiles. An inexhaustible list of end-uses and material classes includes films [2,3,4,5,6], laminates [7,8], and aerogels [9,10]. ACC materials offer a wide range of potential applications, from structural and packing materials to high-value-added engineered electric devices and biomedical purposes [11,12,13,14,15].

One of the great advantages of ACCs is their good interfacial compatibility between the matrix and reinforcement phases, as both are chemically very similar or identical. This allows for efficient stress transfer across the interface area of the composite [11,16,17] and easier handling after the product’s end-of-life, as ACCs can be recycled and composted [11]. ACCs also permit the production of composites with greater reinforcement content (including a high percentage of non-dissolved cellulose fibers) compared to traditional fiber-reinforced composites [18]. The abundance of available cellulose and its favorable properties, such as biodegradability, chemical stability, biocompatibility, and renewability, are some of the primary motivations for its commercial use [19].

The main chemical components of plant-based cellulosic fibers are cellulose, hemicellulose, lignin, pectin, and waxes. The quality and mechanical properties of the fibers vary depending on the plant species, maturity, and growth conditions as well as the fiber extraction and refining procedures used [20,21]. The inconsistent quality of the natural fibers and the presence of hemicelluloses, lignin, and other “impurities” (i.e., non-cellulosic materials) can negatively affect the preparation process and properties of ACC materials [11,22].

The presence of non-cellulosic materials can be avoided by using regenerated cellulose-based fibers, such as lyocell and viscose, which results in ACCs with great consistency and purity [23,24]. Woven textile materials can also create a strong architecture for composite manufacturing [25]. Cellulose nanostructures have recently attracted significant interest in the production of ACCs due to their diverse and unique properties, such as their optical transparency, ability to enhance mechanical performance, and ability to act as oxygen barriers [12,26]. One of the most popular chemical pretreatments in the manufacture of cellulose nanofibers is oxidation mediated by TEMPO (2,2,6,6-tetramethylpiperidine-1-oxyl [27,28]. Table 1 lists and defines the abbreviations and acronyms used in this study.

There are two methods for producing ACCs. In the one-step method, the fiber structure is partly dissolved in the solvent system, and the dissolved part forms the matrix that embeds the undissolved fiber portion. In the two-step method, a reinforcement is combined with a dissolved cellulose source—often nanocellulose or pulp—which is responsible for forming the matrix. Fabricating ACCs requires the dissolution of the cellulose, which can be challenging due to the strong hydrogen-bonding network in cellulose polymers, their high crystallinity, and their insolubility in common solvents [29,30]. The most-used cellulose solvent system is the viscose process, which involves dissolution into carbon disulfide under strong alkaline conditions [31,32]. The downside of this system, as well as many other cellulose solvents, is the lack of comprehensive chemical recovery and recycling, and the toxic by-products that are produced, such as volatile carbon disulfide emissions [22,31]. Consequently, there is strong interest in finding effective, economical, and environmentally friendly chemical processing methods for dissolving cellulose [29,33,34,35].

Aqueous NaOH-urea is a promising solvent for dissolving cellulose and creating ACCs [3,12,36,37,38,39]. The aqueous NaOH-urea solvent system has been found to be a non-toxic, non-volatile, cost-effective, and environmentally friendly way to dissolve cellulose [29]. The chemical and structural interactions between NaOH, cellulose, and urea depend heavily on the dissolution conditions, concentration, and raw materials (e.g., purity, degree of polymerization, free surface area, and physical and chemical features related to chemical reactivity) [40]. Different solvents, production methods, process conditions, and sources of raw materials contribute to the properties of the ACCs produced.

In recent research on ACCs, the focus has often been on the development of multifunctional films [41,42,43,44,45,46,47,48,49,50,51,52]. However, more investigation is needed into constructive laminates, bulk materials, and how different raw material choices and process conditions play a part in the properties of structured materials. In this study, the one-step method was employed using (1) woven textile and (2) a combination of woven textile and TEMPO-oxidized nanocellulose to produce ACCs using aqueous NaOH-urea as a sustainable solvent. To the best of our knowledge, no prior study has investigated lyocell–Spinnova material as a raw material for ACCs. Furthermore, the addition of nanocellulose in refined woven textiles in ACC production needs deeper investigation. A comprehensive analysis of the composites’ thermal and mechanical properties was performed, as these characteristics determine the resulting material’s range of applications. The dissolution time and temperature of hot pressing were also researched.

## 2. Materials and Methods

### 2.1. Materials and Treatments

Sodium hydroxide was mixed with reagent-grade urea and water to prepare the aqueous NaOH-urea solvent. The woven cellulose fabric used in the experiments was provided by Spinnova Ltd., Jyväskylä, Finland. The fabric weighed 185 g/m^2^ and was a plain weave with 2 ply lyocell yarn with 13.2 ends/cm in the warp and 60/40% Spinnova/lyocell yarn with 13.0 picks/cm in the weft. Spinnova’s micro-fibrillated cellulose (MFC), used to produce yarn, is mechanically refined from raw pulp without dissolution or regeneration. According to the manufacturer, the hemicellulose and lignin contents total less than 5%.

To produce the TEMPO-oxidized cellulose nanofibrils (TCNF) used in this study, bleached softwood kraft pulp was oxidized using the well-established TEMPO-mediated oxidation procedure described in detail by Isogai et al. [53]. Briefly, to produce TCNF, bleached softwood kraft pulp was dispersed in deionized water and oxidized with the following reagent loadings: 12.5 mg/g of TEMPO, 125 mg/g of NaBr, and a sodium hypochlorite loading of 5 mmol/g of dry pulp equivalent. Fibrillation of the oxidized pulp was performed on a microfluidizer (M110EH Microfluidizer^®^, Microfluidics, Westwood, MA, USA) by applying one homogenizing step with flow cells APM400 and IXC200 at 1000 bar, followed by three microfluidization passes with APM400 and IXC100 flow cells at 1000 bar. All chemicals used in the production of TCNF and the NaOH solvent system were reagent grade, supplied by Sigma-Aldrich (Darmstadt, Germany), and used as received.

### 2.2. Composite Laminate Preparation

The NaOH-urea solvent was prepared by dissolving NaOH and urea in water to yield a solution with 12 wt.-% NaOH and 7 wt.-% urea. When 0.5 L of a clear solution was obtained, it was chilled to −12 °C and used to prepare the composites. ACC laminates were formed by layering three sheets of woven textile unidirectionally on top of each other. The fabric sizes were 12 × 7.5 cm, and the weight of the resulting samples comprising the three layers was recorded to be approximately 4.5 g. If the layers fell apart during the dissolution or rinsing process, they were manually joined together before pressing. The effects of dissolution time, compression temperature, and the use of TEMPO-oxidized cellulose nanofibrils (TCNF) were investigated in three separate experimental setups. The laminates were formed in a hydraulic hot press (Carvel Press 3581-0, Carver Inc., Wabash, IN, USA).

**Figure 1:**
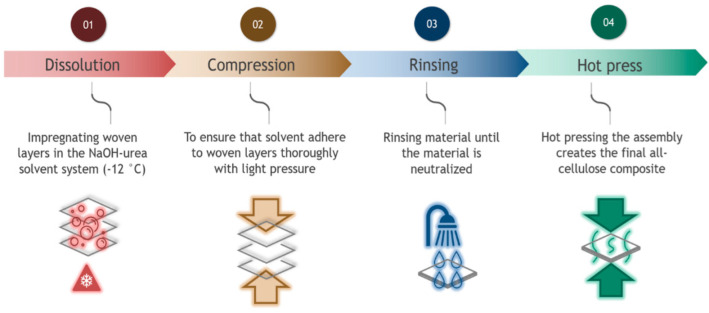
Production steps for the all-cellulose composites (ACCs).

In the first experiment, dissolution times from 2 s to 15 min were used to determine how dissolution time affects the tensile strength of the 3-layer composites. The dissolution phase was followed by pressing under low pressure (1 bar) and solvent removal in a water bath. The water was changed multiple times until the pH of the water and the surface of the composites were neutral. This process took approximately 30 h. Neutralized samples were then cold pressed (1 s under 20 bar) to remove excess water. This was followed by applying hot pressure at 100 °C. For each sample, the pressure in the press was 50 bar and was applied in five steps: first, two short hot press steps of 10 s followed by three additional pressing-release cycles of 1, 3, and 5 min of active time. A one-minute break was used between each step to allow the steam to evaporate. In the last step, all samples were kept for 20 min at 70 °C under a pressure of 10 bar. Figure 1 illustrates the sample preparation process.

In the second experiment, dissolution times were kept constant at 30 s for all samples. Depending on the sample, a hot-pressing temperature between 70 °C and 150 °C was applied. The material preparation followed the conditions listed above.

For the third experiment, a TCNF gel (1 wt.-% solids content) was spread between the woven layers before dissolution treatment with NaOH-urea solvent. Samples were treated using the same press–release program at four different temperatures: 80 °C, 100 °C, 120 °C, and 140 °C. Aside from the addition of TCNF, material preparation followed the conditions mentioned above.

### 2.3. Characterization

#### 2.3.1. Tensile Test

Tensile tests were performed using a Zwick/Roell 050 universal tester (Ulm-Einsingen, Germany). Samples for the tensile test had a length of 100 mm and width of 10 mm. Based on preliminary experiments, samples were tested with a 2.5 kN/mm load cell and a testing speed of 2 mm/min until failure. The setup was chosen because it had been used successfully in earlier studies with similar materials [54]. The average values for each laminate type were calculated from five replicates. All tested composite laminates were measured in the warp (100% lyocell) loading direction.

#### 2.3.2. Thermogravimetric Analysis

Thermogravimetric analysis (TGA) and differential thermogravimetric analysis (DTGA) were conducted using a TA instrument (SDT Q500, New Castle, DE, USA). Samples of approximately 12 mg weight were heated from 30 °C to 650 °C in a nitrogen environment. The nitrogen flow rate was set to 40 mL/min, and the heating gradient was set at 10 °C/min. Four tests for each sample were run to ensure the reproducibility of the results.

#### 2.3.3. Dynamic Mechanical Analysis

Dynamic mechanical analysis (DMA) was performed using a single cantilever test with a Rhemetrics solids analyzer (RSA) II (TA Instruments, New Castle, DE, USA). The temperature range was set from 25 °C to 220 °C, the heating rate was set to 3 °C/min, and the testing frequency was 1 Hz. Test samples were cut to a rectangle shape (50 mm × 10 mm) in a warp yarn (100% lyocell) direction. Four tests were performed for each variant. The temperature dependence properties of storage modulus (*E*′), loss modulus (*E*″), and damping factor (tan *δ*) were analyzed.

#### 2.3.4. Scanning Electron Microscopy

Morphological changes on the surface of the ACCs after different treatments were detected using scanning electron microscope (SEM) imaging carried out on layered ACCs using a Hitachi S-4800 (Hitachi, Tokyo, Japan). The samples were gold coated (2 nm) with a Cressington sputter coater 208 HR (Watford, UK).

#### 2.3.5. Statistical Analysis

Statistical evaluation of the data was completed using one-way analysis of variance (ANOVA) and Tukey–Kramer post hoc analysis, with *p* < 0.05 suggesting significant difference. Test were performed with five replicates per sample.

## 3. Results and Discussion

### 3.1. Tensile Test

#### 3.1.1. Effect of Dissolution Time

**Figure 2:**
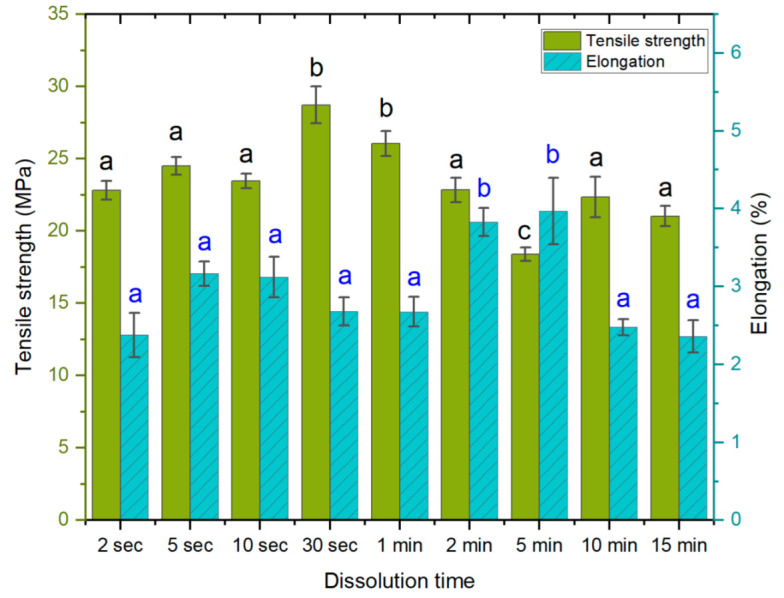
Tensile strength (MPa) and elongation at break (%) as a function of dissolution time. The lettering (black for tensile, blue for elongation) indicates statistical differences between samples divided into groups a, b and c for both properties. The tensile strength of samples with 30 s, 1 min, and 5 min dissolution times differed in comparison to all others. The higher elongation values were noted only in samples with 2 min and 5 min dissolution times in comparison to others.

In the first experiment, different dissolution times were applied, ranging from 2 s to 15 min. The values for tensile strength (Figure 2) were found to be the lowest at a dissolution time of 5 min (18.4 MPa) and the highest at 30 s (28.7 MPa). The elongation at break was the lowest at a dissolution time of 15 min (2.3%) and the highest at 5 min (3.9%). Both the 2 min and 5 min samples exhibited higher elongation at break values, suggesting a reduced interaction between the fibers, which would also result in lower tensile strength. It is possible that longer dissolution times allow the solvent to penetrate deeper into the fibers, causing a disorientation of the weave. Thus, the lower elongation in the subsequent 10 min and 15 min samples and the higher tensile strength could come from a more complete dissolution, followed by homogeneous regeneration. Further research is needed to determine the surface dissolution kinetics of Spinnova fibers. The tensile strengths of the 30 s and 1 min treatments were significantly higher than those of the other samples (*p* < 0.05); similarly, a dissolution time of 5 min provided the weakest samples. Elongation was significantly higher for the 2 min and 5 min samples.

Previous studies have demonstrated that if ACCs are immersed in a solvent, the structure and crystallinity of the cellulose change. When the immersion time in the solvent increases, the crystalline regions disintegrate into amorphous domains as long as the dissolution process is active; thus, longer dissolution times tend to produce ACCs of lower mechanical strength, which is undesirable. Upon the removal of the solvent, the dissolved amorphous regions re-solidify [7,8,55,56]. Changes in the polymer structure, such as the degree of polymerization, tend to correlate to the mechanical properties, as observed by Dormanns et al. [7], who reported a decrease in tensile strength with increasing dissolution times. In this study, the dissolution time yielding the best mechanical properties was relatively short.

In this experiment, the difference between the shortest (2 s) and the longest (15 min) dissolution time was minimal, while the respective tensile strengths were 22.8 MPa and 21 MPa, and the elongations at break (%) were 2.3% for both. The initial dissolution of 30 s produced an increase in the tensile strength of the final ACC. This is likely the result of surface-limited dissolution and the formation of a homogeneous network upon regeneration, with the bulk of the fiber remaining intact and retaining the properties of the source material. As noted, longer dissolution times affect the bulk of the fiber and change the crystalline structure, which is converted into an amorphous state. The likely explanation for this is that the time needed for effective cellulose chain depolymerization is, in fact, longer than the times used in this study. Dry, hydrophilic cellulosic samples will rapidly absorb the NaOH-urea solvent after its addition. In addition, the solvent does not immediately disappear from the laminate when it is washed in water. Instead, dissolution continues for some time until the water around the material neutralizes the solvent. Thus, shorter dissolution times—which produce a lower degree of dissolution of the fiber—would allow for faster regeneration than in cases in which the bulk of the fiber is affected, and the non-solvent (water) needs more time to penetrate deeper into the fiber.

Additionally, as the woven textile sheets were placed on top of each other by hand before dissolution and hot pressing, even small changes in the fiber direction could have played a role in the perceived mechanical properties of the final ACCs. From a production perspective, especially for the handmade process, the main challenge was to obtain an even immersion of the material in short dissolution periods (<10 s).

#### 3.1.2. Effect of Hot Press Temperature

**Figure 3:**
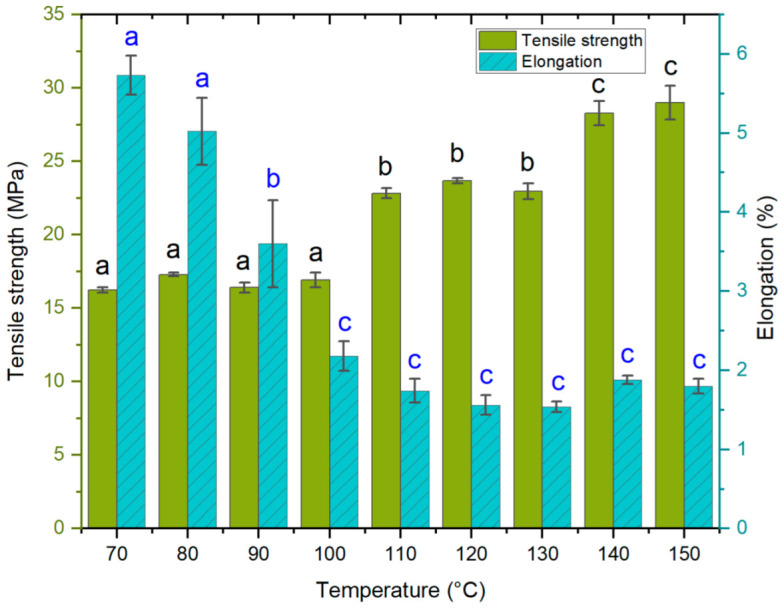
Tensile strength (MPa) and elongation at break (%) (means and standard deviations) as a function of hot press temperature conditions. The lettering (black for tensile, blue for elongation) indicates statistical differences between samples divided into groups a, b and c for both properties. Tensile strength changed in steps, in which samples made ≤ 110 °C (group a) were similar, those made with 110 to 130 °C formed another group (group b), and the highest tensile strength properties (group c) was seen for samples at temperatures of 140 to 150 °C. Conversely, elongation of ACCs was the highest with samples made ≤ 80 °C (group a), with marked decrease already at 90 °C (group b), after which the values plateau (group c).

The second experiment focused on determining how variations in hot press temperature affect the mechanical properties of the laminates. The experiment was carried out by varying the press plate temperatures from 70 °C to 150 °C. Hot press time cycles were the same for each temperature. Based on preliminary tests, a 30 s dissolution time was chosen for all samples. The results showed that a higher temperature during the hot press process increased tensile strength (Figure 3). The change takes place stepwise, not linearly. Temperatures of 70–100 °C, 110–130 °C, and 140–150 °C result in samples with average tensile strength values of approximately 16.7 MPa, 23.1 MPa, and 28.6 MPa, respectively.

The elongation of the composites decreased at higher hot press temperatures. The greatest change occurred when the temperature increased from 70 °C to 100 °C, after which the elongation values remained relatively stable. A minor increase can be seen in the tensile strength and elongation values for 140 °C and 150 °C, compared to the previous values (110 °C, 120 °C, and 130 °C). This may be due to the rapid steaming during hot pressing: when the moisture evaporates rapidly but does not have an easy route to immediate release, the water vapor inside the sample can create extreme pressure. Once the press is opened, the instant development of blisters and voids can occur [57] or a steam explosion can take place. The formation of voids often leads to brittle composites, but they can also be the cause of higher flexibility [58]. One way to limit void formation at this stage is to release steam using short oscillating cycles of pressing.

#### 3.1.3. Effect of Adding TEMPO-Oxidized Cellulose Nanofibrils to ACCs

**Figure 4:**
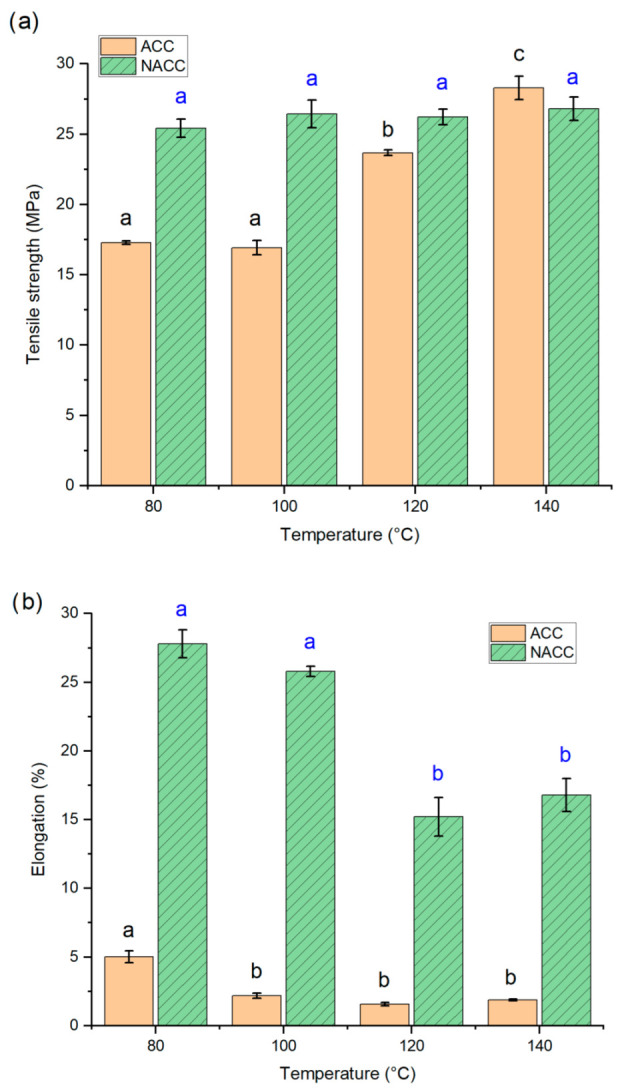
(**a**) Tensile strength (MPa) and (**b**) elongation at break (%) for all-cellulose composites (ACCs) and ACCs with TEMPO-oxidized nanocellulose (NACC) after pressing at different hot press temperatures. The lettering (black for ACC, blue for NACC) indicates the statistical significance of tensile property difference. ACC samples prepared at temperatures of 120 to 140 °C have increased tensile properties, while the NACC sample did not show significant temperature dependency. Both sample types have lowering elongation (**b**); for ACC samples prepared at temperatures higher than 80 °C, this property no longer changes, whereas NACC samples retain their elongation to 100 °C, after which it decreases markedly.

In the third experiment, a small amount of TEMPO-oxidized nanocellulose (1 wt.-% of the composite) was added between the woven textile layers. Samples with TEMPO-oxidized nanocellulose were denominated as NACC. The same dissolution practice and hot press treatment were applied at four different temperatures—80 °C, 100 °C, 120 °C, and 140 °C—as in the previous experiments. The results were compared to laminates produced at the same temperature without added nanocellulose (Figure 4).

Unlike the ACC samples, an increase in hot press temperature did not increase the tensile strength of the NACC samples, as the addition of TEMPO-oxidized nanocellulose achieved maximum effectiveness even at low temperatures. The TEMPO-oxidized nanocellulose acts as an interfacial reinforcement, bridging the gaps between the macroscale fibers and binding them together due to the high surface area, abundance of hydroxyl groups, and high aspect ratio. The mean tensile strength for all NACC samples hot pressed at temperatures between 80 and 140 °C was approximately 26 MPa, which is a higher rate than the ACCs hot pressed at temperatures between 80 and 120 °C. Conversely, tensile strength was slightly lower in the NACCs than in the ACCs when both were hot pressed at 140 °C, but the difference between these was statistically insignificant. This highlights the benefit of adding TEMPO-oxidized nanocellulose to ACCs, as it allows the use of significantly lower pressing temperatures while producing tensile strengths like those of ACCs without added nanocellulose at higher pressing temperatures. The ability to use lower pressing temperatures has a positive impact on energy demand and makes the handling of the material easier and less dangerous. Previous studies have shown that the addition of nanocellulose tends to increase the tensile properties of the composite but also decreases elongation [12,59]. Interestingly, this study found the elongation at break values to be remarkably higher for NACCs than for ACCs. The elongation at break also decreased when the hot press temperature increased. The results indicate that, in the case of NACCs, the elongation at break values are closer to actual lyocell fiber values, which have been reported to be 16–18% for wet and 11–16% for dry fibers [60], and very similar to the value of lyocell-based ACCs of 25.9% (with a 30 min dissolution time in ionic liquid), as reported by Adak and Mukhopahdyay [8].

### 3.2. Thermogravimetric Analysis

A TGA was used to assess the degradation pattern in ACCs and NACCs. Samples were prepared using a dissolution time of 30 s and a hot press temperature of 100 °C.

**Figure 5:**
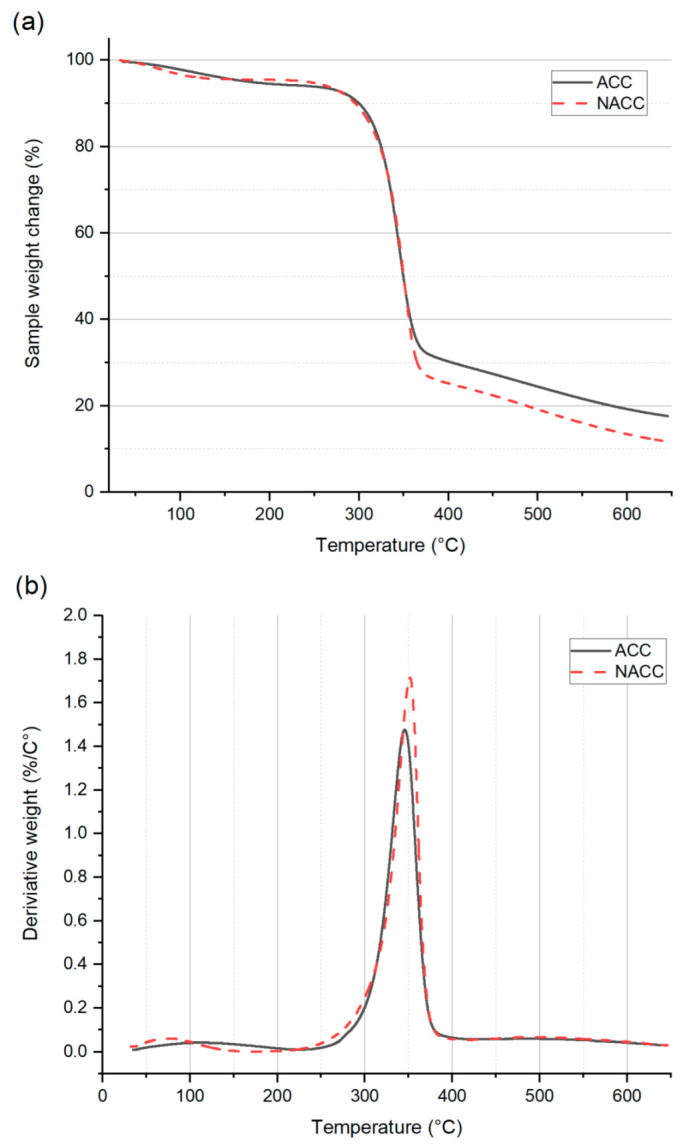
Results of (**a**) thermogravimetric analysis (TGA) and (**b**) differential thermogravimetric analysis (DTGA) for all-cellulose composites (ACCs) and all-cellulose composites with TEMPO-oxidized nanocellulose (NACCs).

A TGA measures the mass loss of the sample as a function of time and temperature. When the materials are heated, the volatile organic matter in the polymer is emitted, which is detected during the analysis [61]. Since the ACCs and NACCs were both formed from cellulose, a marked variation between results was not expected. Some variation could be observed due to the presence of aldehyde and keto groups in the TEMPO-oxidized cellulose, as these groups are known to cause the autohydrolysis of TEMPO-oxidized cellulose in dry material at elevated temperatures [62]. However, the amounts of TEMPO-oxidized cellulose used here were so low that a meaningful contribution was not expected. The TGA curves of the composite samples are shown in Figure 5.

Both the ACCs and NACCs had an initial decrease in mass loss before 100 °C due to the evaporation of moisture. Both samples had rapid, identical decomposition steps between 300 and 360 °C. These results agree with those of previous studies [63,64]. According to the DTGA results, the highest peaks for the derivative weight (%/°C) were at 345 °C for the ACCs and 351 °C for the NACCs. After 360 °C, the NACCs had greater mass loss than the ACCs. This may be the contribution of the TEMPO-oxidized cellulose, as it is known to be thermally less stable than pure cellulose due to the presence of aldehyde and keto groups [65,66]. The trends of the samples remained similar until the end of the heating cycle (645 °C), after which the weight of the sample residue was measured to be 17.6% for the ACCs and 11.7% for the NACCs. These results demonstrate that adding TEMPO-oxidized nanocellulose to ACCs has a negative influence on the composite’s thermal stability. A previous study reported a similar outcome [66], and the effect was attributed to cellulose with a high carboxyl-group content [53]. Deprotonated carboxylic groups, such as those resulting from TEMPO oxidation, can disturb hydrogen bonds and cause less-constrained molecular chains that decrease the thermal stability of the composite [66,67]. It is possible that this adverse impact could be mitigated using phosphorylated nanocelluloses [68].

### 3.3. Dynamic Mechanical Analysis

**Figure 6:**
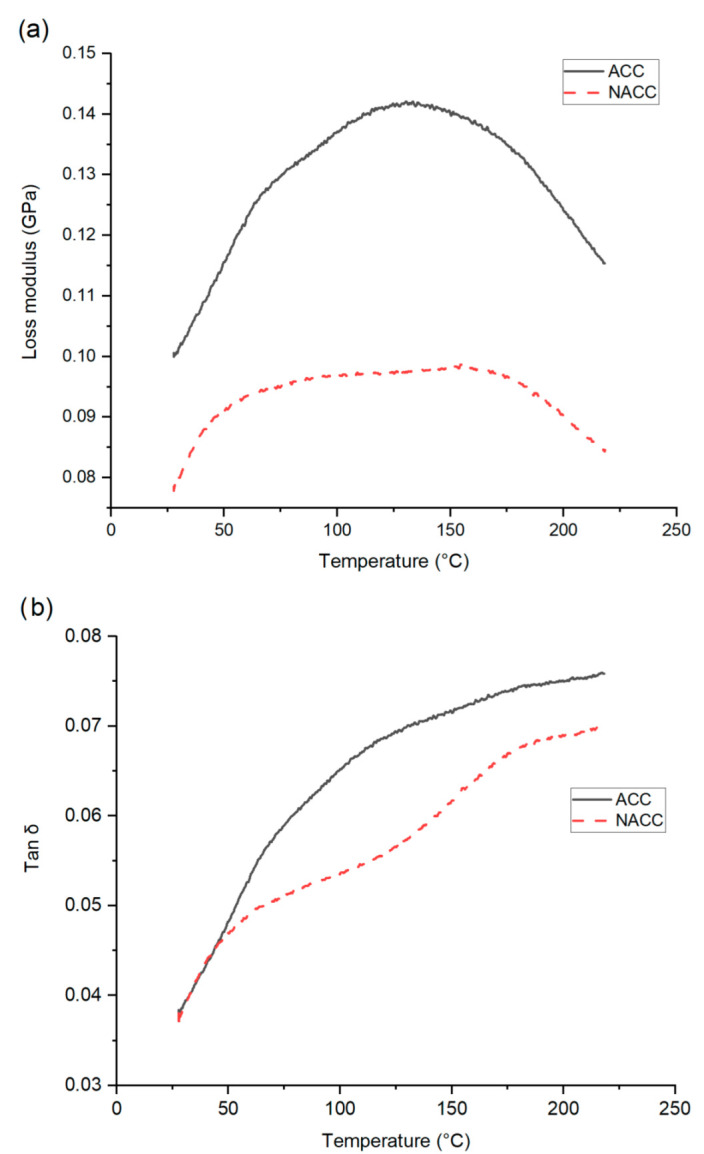
Results of (**a**) dynamic mechanical analysis (DMA) presenting storage modulus and loss modulus and (**b**) tan δ for all-cellulose composites (ACCs) and nanocellulose-doped all-cellulose composites (NACCs).

A DMA of the composite sheets was performed on both the ACC and NACC materials, made with a 30 s dissolution time and pressed at 100 °C. The viscoelastic response is shown in Figure 6. The storage modulus—the energy stored in the elastic deformation phase of a viscoelastic material—was higher for the ACCs and lower (by approximately 0.6 GPa) for the NACCs. The previous literature states that nanocellulose crystallites and nanofibers, as reinforcing agents, can improve the properties of a composite [69], and nanocellulose increases the storage modulus [70,71]. Figure 4 shows that the NACC samples had better tensile properties than the ACC samples. In addition, the elastic properties (elongation before break, %) of the laminates were remarkably enhanced, which may explain why the storage modulus was lower for the NACC samples than the ACC ones. We assumed that the interfacial bond between the reinforcement and matrix was stronger in the ACCs than in the NACCs. Given that ACCs are formed using one identical source of cellulose while NACCs have two different sources of cellulose, it is possible that the cellulose in the NACC samples did not have seamless compatibility. The lack of a tight structure and a packing order for the polymer chains allows the polymers to move, which results in enhanced mobility and increased flexibility.

The storage modulus trend for the ACCs and NACCs was similar: the storage modulus decreased linearly as a function of press temperature. This is a common trend shared by all-cellulose materials [63]. The loss modulus (the energy dissipation associated with the motion of polymer chains) and tan delta (δ) (ratio of loss modulus and storage modulus of polymer composites within the measured temperature range) were both higher for the ACCs. For both the ACCs and NACCs, the tan δ curve showed an upward trend, and neither the ACCs nor the NACCs had a clear peak to indicate the glass transition temperature. Cellulose, like other polymers, undergoes a phase transition from a low-temperature glass state to a higher-temperature elastic state, but the transformation stage is not easily detectable for cellulose [72,73,74]. The results of this study agree with previously identified phenomena [72,73,74].

### 3.4. Scanning Electron Microscopy

**Figure 7:**
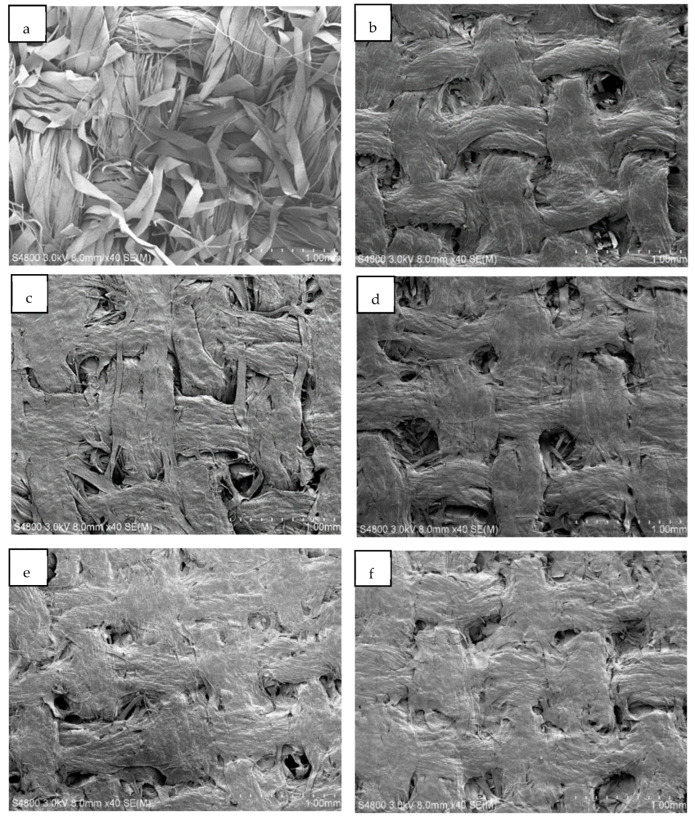
Scanning electron microscope (SEM) images of textile-based ACC laminates. Image (**a**) shows the original, untreated textile, and the rest of the images are of samples produced at specific temperatures: (**b**) 70 °C, (**c**) 90 °C, (**d**) 110 °C, (**e**) 130 °C, and (**f**) 150 °C.

Scanning electron microscopy (SEM) images of original textiles and ACC laminates with hot press temperatures of 70–150 °C are shown in Figure 7. The images show a clear difference between the untreated textiles (Figure 7a) and the three-layer laminates (Figure 7b–f). The fiber structure is transformed during the NaOH-urea dissolution process, and individual fibers are fused together with surrounding fibers during dissolution, forming a more uniform surface. Similarly, Huber et al. [54] observed that flax-textile-based ACCs (formed using ionic liquids with an 80 min dissolution time at 100 °C) had a clear dissolution and regeneration of the fiber surfaces, resulting in the bonding of individual fibers within the yarn.

Figure 7 shows that individual fiber threads are less visible in ACC materials produced at higher temperatures (Figure 7e,f) than in the ones produced at 70 °C (Figure 7b). Nevertheless, ACC laminates produced at higher temperatures also have uneven and porous surfaces. It may be that the NaOH/urea solvent system is not effective enough to dissolve fiber further or it needs a longer dissolution time. Han and Yan [58] discovered that a fine web-like and highly fibrous network was apparent before dissolution, and after one hour of dissolution, the fibers were partially disentangled and dissolved. However, the pores remained on the surface of the composite after two hours of dissolution. It was not until 12 h of dissolution that the treated surface had a high level of homogeneity.

**Figure 8:**
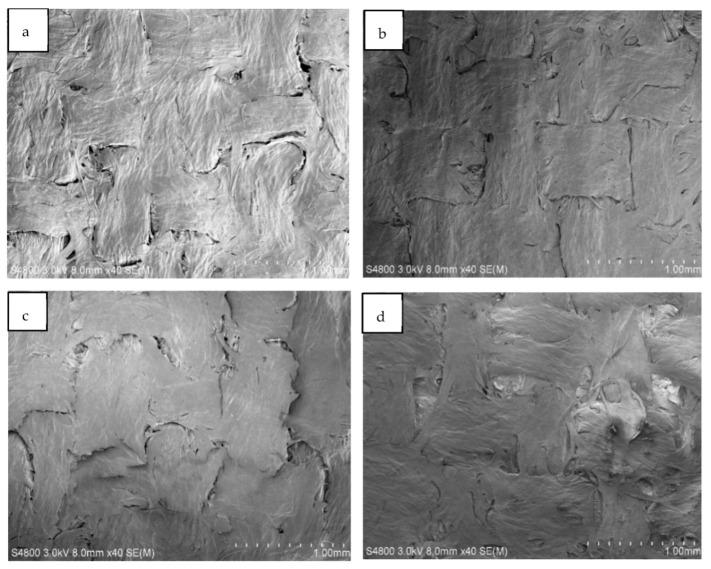
Scanning electron microscope (SEM) images of textile-based NACC laminates. The samples were produced at specific temperatures: (**a**) 80 °C, (**b**) 100 °C, (**c**) 120 °C, and (**d**) 140 °C.

Figure 8 shows SEM images (Figure 8a–d) for NACC laminates made at hot press temperatures of 80–140 °C. Here, the visible difference between production temperature results is limited, although it appears that higher press temperatures yield less apparent interfaces between textile filaments in the micro-scale texture of the ACCs. When comparing ACCs and NACCs, it is clear that NACC laminates produce a smoother surface than ACCs fabricated only with cellulose textiles (Figure 7).

**Figure 9:**
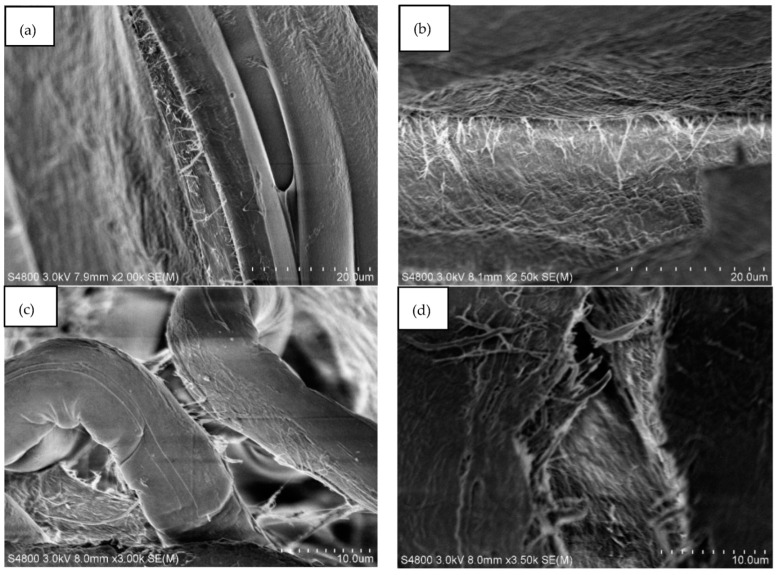
Scanning electron microscope (SEM) images of NACCs produced at a hot press temperature of 100 °C at the following magnifications: (**a**) 2000× (**b**) 2500×, (**c**) 3000×, and (**d**) 3500×.

Figure 9 shows close-up (2000–3500 times magnification) images of the NACC sample with a production temperature of 100 °C. The images make it clear that there are also fibrils bridging the microscale structures. These fibrils are most likely a combination of lyocell–Spinnova fiber surface defibrillation and deposited TCNF. All samples, whether ACC or NACC laminates, had similar fibrillar cross-linking structures. Such interfacial bridging is likely to enhance the mechanical properties of the composites.

## 4. Conclusions

ACCs were successfully produced from layered cellulose-based textiles using a sustainable NaOH-urea solvent system. In addition, nanocellulose-enriched ACC (NACC) samples were produced to study the effects of TEMPO-oxidization. Increased processing temperature in the ACC sample increased the tensile strength and decreased the elongation at break. An increase in processing temperature did not have the same effect on the properties of the NACCs. Adding TEMPO-oxidized nanocellulose made the tensile strength of the NACCs greater than that of the ACCs, especially at low pressing temperatures (80 °C). At higher temperatures (140 °C), the tensile strength benefit of adding TEMPO-oxidized nanocellulose was not observed. The addition of TEMPO-oxidized nanocellulose also greatly increased the elongation at break of the composites, while no improvement was seen on the interface between the reinforcement and matrix or in the interfacial compatibility of NACC composites. Single polymer composites using only lyocell–Spinnova woven material were observed to have a higher storage modulus and better thermal stabilization without the addition of TEMPO-oxidized nanocellulose. The SEM images showed major morphological changes in the surface structure of the woven material after NaOH-urea treatment. Small changes seen in the SEM images show the results of various processing temperatures. At higher press temperatures, the surface of the composite seemed to have a slightly higher uniformity than the surface of the ACCs produced at lower temperatures. However, all samples retained an uneven and porous general structure.

## Figures and Tables

**Table 1:** List of abbreviations and acronyms used in this article.

Abbreviation	Explanation
ACC	All-Cellulose Composite
ANOVA	Analysis of Variance
DMA	Dynamic mechanical analysis
DTGA	Differential thermogravimetric analysis
NACC	Samples with TEMPO-oxidized nanocellulose
NaOH	Sodium hydroxide
SEM	Scanning Electron Microscope
TCNF	TEMPO-oxidized cellulose nanofibrils
TEMPO-oxidation	2,2,6,6-tetramethylpiperidine-1-oxyl -mediated oxidation
TGA	Thermogravimetric analysis

## Data Availability

The data presented in this study are available on request from the corresponding author.
